# Challenges and Opportunities of Public Health Professionals in Nepal: A Reflexive Thematic Analysis

**DOI:** 10.1002/puh2.70327

**Published:** 2026-08-26

**Authors:** Ankit Acharya, Romika Neupane, Bibekti Nepal, Liberty Shrestha, Princy Bhatta

**Affiliations:** ^1^ Department of Public Health Nobel College Pokhara University Kathmandu Nepal; ^2^ Research Division Sustainable Public Health Bhaktapur Nepal

**Keywords:** challenges, education, employment, opportunities, public health

## Abstract

**Background:**

Public health professionals (PHPs) constitute a critical component of health systems, but their ability to apply knowledge effectively across sectors is often constrained. Persistent issues such as inadequate training, limited career opportunities, and job dissatisfaction undermine workforce effectiveness and retention globally. In Nepal, these challenges are compounded by weak alignment between academic preparation and professional realities, creating barriers for graduates and professionals to thrive within the health system.

**Methods:**

Qualitative reflexive thematic analysis (RTA) was used to understand the educational and career experiences of PHPs in Nepal. Guided by principles of information power, we recruited 18 participants via purposive and snowball sampling, and semi‐structured interviews were conducted.

**Results:**

Through reflexive engagement, we constructed six key themes: (1) navigating a narrow job market, (2) struggling with practical gaps, (3) barriers to job placement, (4) foundations misaligned with practice, (5) overcoming challenges, and (6) hope and future vision. The participants reported a saturated and uncertain job market, limited mentorship, inadequate practical training, nepotistic recruitment practices, and curricula misaligned with contemporary public health practices. These factors constrain employability and career satisfaction, often prompting additional study or career shifts. Despite systemic barriers, participants expressed cautious optimism that Medical Education Commission (MEC)‐led reforms, including standardized entrance examinations and integrated scholarships, together with potential vacancies at local government levels, could enhance education, improve workforce retention, and create meaningful professional roles in Nepal.

**Conclusions:**

This study highlights the urgent need to realign Nepal's public health education with professional realities. Strengthening curricula with practical, competency‐based training, integrating modern technologies, providing mentorship and field exposure, and ensuring transparent employment and supportive policies can empower public health graduates and professionals. Such reforms enhance workforce resilience, career satisfaction, and professional identity, ultimately strengthening public health capacity to improve population health outcomes in resource‐constrained contexts.

## Graphical abstract

Behind Nepal's growing public health workforce lies a critical disconnect between training and real‐world practice, shaping career trajectories and system capacity. This study reveals how structural barriers and emerging opportunities are redefining professional pathways—offering urgent insights for transforming education, employment, and the future of public health.

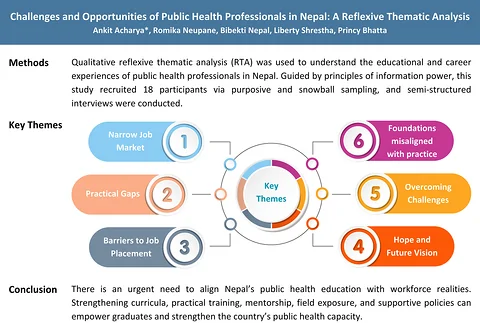

## 1 Introduction

Public health professionals (PHPs) constitute the backbone of health systems worldwide, addressing complex population‐level challenges through the assessment of health problems, prevention and control of diseases, health promotion, policy development, and community engagement [1, 2, 3]. Their roles encompass a wide array of responsibilities, from managing public health emergencies to promoting health equity, yet they often face significant barriers in translating knowledge into practice across sectors and systems [4, 5]. In an era marked by evolving threats such as climate change, pandemics, and digital transformations, the significance of a well‐prepared public health workforce has never been more pronounced [6, 7, 8]. However, global reviews highlight persistent challenges, including inadequate training, job dissatisfaction, and limited opportunities for professional development, which undermine workforce effectiveness and retention [9, 10, 11].

Public health workforce research further identifies persistent structural constraints within health systems. Trained professionals are frequently underutilized and inconsistently positioned across institutional settings. Variability in job classifications, role expectations, and deployment practices limits their effective contribution to population health outcomes. These limitations are compounded by ongoing challenges in recruitment and retention, alongside insufficient data on workforce composition, which hinder strategic workforce planning [12]. These constraints are exacerbated by ongoing transformations in global health governance. Globalization, digitization, and commitments to the Sustainable Development Goals have expanded the scope of public health practice to multi‐level interventions. As a result, the demand for adaptive, interdisciplinary, and practice‐oriented competencies has increased substantially, particularly in areas such as health literacy, digital health, risk communication, and stakeholder co‐creation [13].

The education and training of public health students and professionals are pivotal in assisting them in navigating these demands. Over the past two decades, undergraduate and postgraduate public health programs in Nepal and elsewhere have expanded significantly, emphasizing competencies in areas such as interprofessional collaboration, leadership, and emerging technologies such as artificial intelligence [14, 15, 16, 17, 18]. Despite these advancements, significant gaps remain between public health curricula and the competencies required in real‐world practice. In particular, existing programs often inadequately address skills related to advocacy, social change, cross‐cultural competence, and multidisciplinary and transdisciplinary collaboration [19, 20, 21, 22, 23, 24]. Employers increasingly prioritize practical abilities, such as data management and research skills, project management, and community engagements, yet many professionals report mismatches between their training and job requirements [25, 26, 27]. In high‐income contexts, initiatives such as self‐directed online learning and reflective practices have shown promise in enhancing career readiness [28, 29], but these initiatives are often underutilized in resource‐constrained settings such as Nepal [30]. This gap is further compounded by traditional educational norms prevalent in the South Asian context, where hierarchical faculty‐student relationships and an emphasis on rote learning often dominate. Such practices are poorly aligned with the needs of public health education, which thrives on open dialogue, interdisciplinary collaboration, curiosity, and creative problem‐solving [24].

Emerging evidence, although comparatively limited, highlights several opportunities to strengthen the public health workforce that may help mitigate persistent structural and capacity‐related challenges. Studies indicate that public health roles are inherently attractive to early‐career professionals due to their mission‐driven nature, opportunities for meaningful community impact, job security, and potential for professional growth, suggesting a strong motivational base that can be leveraged for recruitment and retention [31]. At the same time, the rapid expansion of public health education and the growing pool of graduates present an underutilized pipeline that, if better aligned with governmental and practice settings through structured partnerships, internships, and clearer career pathways, could address workforce shortages and skill mismatches [31, 32]. Evidence further shows that early‐career professionals, many entering from diverse sectors, bring valuable interdisciplinary skills and demonstrate a willingness to remain in the field, particularly when supported through leadership development, mentorship, and transparent advancement opportunities [32]. Additionally, system‐level improvements—such as modernizing recruitment processes, enhancing transparency in job roles and compensation, fostering innovative and inclusive organizational cultures, and strengthening academia and practice linkages—have been identified as actionable strategies to improve workforce integration and retention [31]. Collectively, these opportunities suggest that targeted investments in recruitment, career pathways, and organizational reform can help bridge existing gaps between training and employment, enhance workforce sustainability, and improve the overall effectiveness of public health systems.

The institutionalization of public health in Nepal accelerated after the establishment of the Ministry of Health in 1956 and the launch of national disease control programs, including malaria eradication and smallpox control initiatives. Formal public health education began with the establishment of the Institute of Medicine under Tribhuvan University in 1972, followed by the introduction of the bachelor of public health (BPH) program in 1986 and postgraduate training in the early 1990s. As of 2025, nearly 10,000 PHPs are registered with the Nepal Health Professional Council (NHPC), including over 7800 general‐level and approximately 1900 specialist‐level practitioners [33]. This growth reflects the progressive institutionalization of public health as a recognized professional field in Nepal.

In Nepal, the Medical Education Commission (MEC), established under the National Medical Education Act 2075, plays a central role in regulating and advancing medical education across all health professions. In 2025, MEC allocated 670 BPH seats across 21 private and 9 public institutions, with 201 reserved for scholarships distributed across categories such as open merit, female, Dalit, Dalit female, Muslim, Muslim female, Adibasi Janajati, Khas Arya, Madhesi, Madhesi Dalit, Tharu, students from disadvantaged areas, disabled students, and martyrs’/missing family members. At the postgraduate level, 179 master of public health (MPH) seats were available, including 91 in 6 public institutions and 88 in 10 private institutions. Together, a total of 849 students were expected to pursue public health education in Nepal in 2025 [34]. In 2014, 26 institutions offered undergraduate public health education, with a total annual intake of 1000 students, whereas four postgraduate programs collectively admitted 49 students per year [35]. On the one hand, the reduced number of undergraduate seats reflects the regulation of public health education in Nepal, whereas on the other hand, it also shows how inadequate institutional capacities exist. Simultaneously, it underscores the growing need for a well‐trained public health workforce capable of responding to evolving health challenges. In this context, MEC plays a critical role in ensuring that public health education aligns with national health priorities while promoting professionalism, accountability, and equitable access. These dynamics frame the ongoing debate about the capacity and direction of public health education in Nepal and highlight the importance of aligning academic preparation with the needs of the health system.

Studies from developing countries, such as Ethiopia, Pakistan, India, and Nepal, reveal low job satisfaction among PHPs due to factors such as organizational restructuring, poor remuneration, limited career progression, and inadequate support for interdisciplinary education [36, 37, 38, 39, 40, 41]. The Eastern Mediterranean and Asian contexts further illustrate innovative approaches to training but underscore the need for context‐specific strategies to address public health challenges and ecosystem changes [42, 43, 44]. In addition to these challenges, opportunities emerge, such as integrating arts, human rights, and One Health principles into education to build resilient professionals [1, 45, 46, 47].

Nepal, as a developing country in South Asia, exemplifies these dynamics, with its public health sector struggling with rapid urbanization, natural disasters, and a growing burden of noncommunicable diseases. However, research on the experiences of PHPs in Nepal remains limited, with most evidence drawn from broader regional or global studies that may not capture local contexts [48, 49, 50]. This gap prevents targeted interventions to improve workforce outcomes and strengthen the health system. To address this, the present qualitative study explores the challenges and opportunities faced by PHPs in Nepal, drawing on in‐depth interviews to provide insights into their professional trajectories, skill gaps, and aspirations. By illuminating these perspectives, this research aims to inform educational reforms, policy enhancements, and strategies for fostering a robust public health workforce in Nepal and similar contexts, ultimately contributing to global efforts in public health equity and preparedness.

## 2 Materials and Methods

### 2.1 Design

This study employed a reflexive thematic analysis (RTA) approach with a qualitative design to explore the lived experiences of PHPs in Nepal, with a particular emphasis on the challenges and opportunities shaping their educational and professional trajectories. RTA was selected for its interpretive flexibility, enabling the construction of rich, contextualized patterns of meaning from participants' perspectives that align with the study's exploratory aims [51]. Unlike approaches emphasizing coding reliability or preconceived categories, RTAs foreground the researcher's active role in knowledge production through reflexive engagement with the data, prioritizing depth of insight over replicability [51]. This study draws on the RTA Reporting Guidelines (RTARG) to ensure transparency and methodological coherence [52].

### 2.2 Study Setting and Participants

The research was conducted in Nepal, encompassing PHPs employed across diverse sectors, including government health facilities, nongovernmental organizations (NGOs), academic institutions, and private entities. To be eligible, participants needed to have completed a bachelor's or master's degree in public health from any recognized university within the past 5 years, hold valid registration with the NHPC, and be either enrolled in an MPH program, employed in a public health role, or seeking employment in the public health sector.

Purposive sampling was employed to select participants on the basis of their relevance to the research questions and potential to provide in‐depth perspectives, ensuring maximum variation in gender, years of professional experience, educational level, employment sector, and geographical location [53]. This was supplemented by snowball sampling through participant referrals to access eligible individuals, as they were often better positioned than institutions to identify peers or colleagues who were currently working in public health, studying, or seeking employment. An initial phone call was made to the referred participants to explain the study and ask for their consent, and if obtained, they were scheduled for a phone call or Zoom meetings interview. Recruitment continued until thematic saturation was achieved, defined as the point at which no new themes emerged from additional data, guided by principles of information power emphasizing data richness over sample size [54]. No benefits of participation were provided to the participants.

### 2.3 Theoretical Assumptions

Prior to data collection and analysis, the research team explicitly addressed key theoretical assumptions to guide the RTA process, following Braun and Clarke's framework of four continua [51]. A constructionist epistemology was adopted, viewing participants’ experiences as socially and discursively produced rather than direct reflections of objective reality, enabling an exploration of how PHPs construct meaning around their trajectories within Nepal's sociocultural and professional contexts [55]. The analysis took an experiential orientation, centering participants’ subjective perception of their educational and career pathways while incorporating critical elements to examine broader power dynamics, including sectoral inequalities and policy influences. A predominantly inductive approach allowed themes to emerge organically from the data, with selective deductive input to ensure relevance to the research questions, such as linking emergent patterns to known barriers in public health education. These assumptions were continually revisited through team discussions to reflect on how they shaped interpretations throughout the analytic process.

### 2.4 Data Collection

Data were collected through semi‐structured, in‐depth interviews conducted between January and June 2023. The interviews were held either in person or through a secure video conferencing platform, Zoom meetings, to accommodate participants' preferences and logistical constraints. Each interview lasted 30–45 min and was audio‐recorded with explicit participant consent. Given that interviews were conducted remotely (Zoom meetings or telephone), verbal informed consent was obtained and audio‐recorded at the start of each interview, before data collection began. Participants were provided with a clear explanation of the study's aims, procedures, potential risks, anticipated benefits, and their rights before consenting.

To minimize risk of harm, the research team planned that, should a participant show signs of discomfort or distress, the interview would be paused, and participants would be reminded of their right to discontinue participation. Participants were also informed that they could request assistance at any time, such as guidance in identifying local counseling, mental health, or emergency support services if needed during or after the interview.

The interview guide was informed by a literature review and pilot tested with three non‐participating individuals to enhance question clarity, relevance, and flow. Probing questions were used flexibly to encourage elaboration while maintaining focus. The interviews were conducted by researchers in Nepali or English, who were bilingual and had moderate to excellent experiences in qualitative interviewing, based on participant preference. No prior relationships existed between the interviewer and interviewee to minimize potential bias.

### 2.5 Data Analysis

Audio recordings were transcribed and translated into English, with accuracy checked through careful review. The analysis was conducted via Braun and Clarke's RTA framework, which was chosen for its flexibility, attention to researcher subjectivity, and fit with the study's exploratory aims [51]. We adopted an inductive orientation, allowing codes and themes to be developed from the data rather than applying predefined categories. In keeping with the principles of RTA, themes were understood as patterns of shared meaning constructed through reflexive engagement with the dataset rather than as entities that simply “emerged” from the data.

The analytic process was iterative and recursive, moving across Braun and Clarke's six phases: (1) familiarization through repeated reading and immersion in the dataset; (2) systematic coding to identify semantic/surface and latent/conceptual segments, generating initial codes; (3) generating initial themes; (4) developing and reviewing themes; (5) refining, defining, and naming themes to capture their essence; and (6) producing the analytic narrative [51]. Coding was exploratory in the early stages, with all potentially relevant meanings kept in play before moving toward more interpretive patterning. NVivo and MS Excel were used pragmatically to support coding, organization, and visual mapping.

Coding served as the foundational step, with each code representing a succinct analytic idea capturing a specific meaning relevant to the research question. Transcripts were read multiple times, and initial codes were generated inductively, reflecting semantic and latent meanings. Codes were applied to discrete segments of data, ensuring that each captured a single, coherent meaning, whereas multiple codes were applied to the same segment where relevant. Early coding was exploratory, with all potentially meaningful ideas retained to allow for the discovery of patterns. Two researchers (A.A. and P.B.) independently coded a subset of transcripts, comparing and refining codes through discussion to form a preliminary coding framework. This framework was then iteratively applied across the dataset, with new codes added as fresh insights emerged and existing codes refined to better differentiate meanings.

Candidate themes were generated by clustering related codes around central concepts that captured shared patterns of meaning rather than simply summarizing topic content. Visual mapping techniques were employed to explore relationships among codes and identify clusters indicative of emerging themes. Each potential theme was elaborated through written summaries to clarify its scope, boundaries, and conceptual essence. Themes were iteratively reviewed against the dataset to ensure coherence, relevance, and richness while avoiding over‐fragmentation or dilution of meaning. The final themes were codeveloped collaboratively through reflective discussions within the research team (A.A., R.N., B.N., L.S., and P.B.), ensuring interpretive depth, consistency, and a clear narrative linking the analytic insights to the study's objectives. This approach emphasized the journey of analysis—tracking how codes evolved into coherent themes—rather than prematurely finalizing categories, thereby ensuring that the analytic narrative accurately represented the data while highlighting participants’ perspectives.

### 2.6 Trustworthiness and Reflexivity

Consistent with best practices in RTA, the research team attended to relational ethics and reflexivity across the research process, including consideration of power dynamics during interviews and the potential implications of researcher positionality for data generation and interpretation. Throughout the study, the research team engaged in reflexive discussions to recognize how their professional backgrounds and positionalities shaped the research process. All members of the research team are PHPs, providing diverse expertise to the study. A.A., a poet and health economist; R.N., a physical trainer with a nursing background; and early‐career researchers B.N., a yoga trainer, and L.S., a sociologist, contributed perspectives shaped by their academic training and field experiences in Nepal. P.B. provided practical insights from program implementation and policy advocacy, adding an applied lens to the analysis of workforce challenges. The team acknowledged that their shared grounding in public health education in Nepal could influence both data interpretation and sensitivity to participants’ perspectives. To mitigate these influences and enhance reflexivity, team members regularly engaged in critical discussions, questioned assumptions, and explored alternative interpretations during data analysis and theme development.

### 2.7 Ethical Considerations

The study received ethical approval from the Institutional Review Committee of Nobel College, Kathmandu, Nepal (Approval No. EPHIRC036/2022). All procedures complied with the Declaration of Helsinki [56]. The consent process, including verbal consent and audio documentation, was undertaken in accordance with Nepal Health Research Council guidance for ethical conduct of health research [57]. Consent included permission to record interviews and to publish anonymized quotations in academic outputs.

Participation was entirely voluntary. Participants were informed that they could decline to answer any question, pause or stop the interview, and withdraw from the study at any time without penalty.

Confidentiality was emphasized throughout. No identifying information was retained in transcripts or reports. Transcripts were anonymized and stored separately from any contact details, and participants were assigned pseudonyms. To minimize the risk of deductive disclosure when presenting qualitative excerpts, potentially identifying contextual details were removed or altered where necessary without changing the substantive meaning of participants’ accounts.

All data were stored in a secure, encrypted Google Drive folder with access strictly limited to the research team.

## 3 Findings

A total of 18 IDIs were conducted through phone calls (*n* = 8) or Zoom meetings (*n* = 10), ranging in age from 23 to 32 years (mean 27 years). All participants held a valid license from the NHPC and had either a BPH (*n* = 9) or an MPH (*n* = 9) as their highest qualification, with professional experience spanning 1–5 years since their last degree. At the time of data collection, participants were engaged in diverse roles, including government positions (*n* = 4), NGO or international organizations (INGO; *n* = 4), academic institutions (*n* = 2), private hospitals (*n* = 1), MPH studies (*n* = 4), or were seeking employment at the time of data collection (*n* = 3). The sociodemographic characteristics of the participants are summarized in Table 1.

**TABLE 1:** Demographic characteristics of IDI participants (*n* = 18).

ID	Age (years)	Gender	Highest degree	Years of experience	Education (MPH)/employment (NGO/INGO)/seeking employment
P1	24	Female	BPH	1	NGO/INGO
P2	27	Male	MPH	3	Government
P3	29	Female	MPH	3	NGO/INGO
P4	32	Male	MPH	4	Student
P5	26	Female	BPH	2	Seeking employment
P6	25	Male	BPH	3	Government
P7	28	Female	MPH	4	Student
P8	26	Male	BPH	2	Academia
P9	25	Female	BPH	2.5	NGO/INGO
P10	30	Male	MPH	3	Student
P11	24	Female	BPH	3	Government
P12	27	Male	BPH	3	Private hospital
P13	26	Female	MPH	2	Student
P14	23	Male	BPH	1	Seeking employment
P15	27	Female	MPH	2.5	NGO/INGO
P16	28	Male	MPH	1	Seeking employment
P17	30	Female	MPH	5	Government
P18	27	Male	BPH	4	Academia

Abbreviations: BPH, bachelor of public health; INGO, international nongovernmental organization; MPH, master of public health; NGO, nongovernmental organization.

The following themes and subthemes were generated on the basis of the RTA of the data, as suggested by V. Braun and V. Clarke [58]:

1. **Navigating a narrow job market**
2. **Struggling with practical gaps**
3. **Barriers to job placement**
4. **Foundations misaligned with practice**
5. **Overcoming the challenges**
6. **Hope and future vision**

A brief summary of the themes, subthemes, sample illustrative quotes, and analytic interpretations is presented in Table 2.

**TABLE 2:** Summary of themes, subthemes, sample illustrative quotes, and analytic interpretation.

Theme	Subthemes	Illustrative quotes	Analytic interpretation
**1. Navigating a narrow job market**	Job scarcity and uncertainty; competition from higher qualified candidates; further studies for employability; proactive skill development	“Owing to difficulty in job, people prefer joining masters … reason behind joining masters is to get a job easily.”—P6 “Opportunities exist … but … if 100 students graduate every year, many may remain without a job.”—P1	Professionals perceive the labor market as saturated and uncertain. Postgraduate study is often a strategic response to improve employability rather than purely academic interest. Scarcity influences career trajectories and professional identity formation
**2. Struggling with practical gaps**	Theory‐practice disconnect, inadequate mentorship culture, influence of social capital, independent skill bridging	“During our study, we focused mostly on books and assignments … when I joined a local NGO, I realized that I did not know how to manage a public health project … universities say we are ready, but reality is different.”—P9	Transitioning from university to work is challenging. Participants struggle with applying theoretical knowledge in practice. Limited mentorship, guidance, and practical exposure exacerbate feelings of uncertainty and hinder skill development
		“After graduation, I didn't know where to begin. With mentorship from someone to guide me … on skill development and job search, I would have felt more confident. Instead, I felt lost ….”—P18	
**3. Barriers to job placement**	Limited opportunities, disparities in opportunities, unfair recruiting, and demoralization among workforce	“Competent candidates miss out on job opportunities owing to limited vacancies and unfair recruiting, as employers often prioritize their acquaintances.”—P16	Structural and systemic factors affect placement. Political and personal connections often outweigh merit. Internships and job opportunities are limited or inconsistent, leaving professionals underprepared and anxious about employment prospects, ultimately leading to dissatisfaction in the field
		“Even after graduation, you constantly feel stressed …. I was lucky to find a job when some of my friends were even thinking about leaving public health entirely … waiting for job is so demoralizing.”—P15	
**4. Foundations misaligned with practice**	Unfair recruiting, outdated curricula, regulatory gaps, education‐practice disconnect	“We have NHPC for quality control. They conduct licensing exams, where only around half of the students pass … the question arises about students’ competencies … competencies come from universities, and finally, the question arises about universities themselves—whether a university is providing the right education and training.”—P1	Educational institutions contribute to skill gaps. Curricula emphasize theoretical learning, rarely updated with real‐world practices or technologies. Professionals often leave with broad but shallow knowledge, insufficient for professional qualifications and practical job demands
		“The syllabus has not changed for decades … we were not prepared for current field realities because the curriculum lagged behind actual public health practice.”—P13	
**5. Overcoming the challenges**	Dedicated public health posts, updated curriculum, competency‐based standards, systemic reform, and advocacy	“The government should allocate specific posts for public health graduates at the Palika level. This will reduce overreliance on other health cadres and ensure that graduates’ skills are fully utilized in community health management.”—P14	Professionals identify gaps in workforce deployment and education, emphasizing the need for designated public health posts at the Palika level, regularly updated field‐relevant curricula, and actionable strategies—including experiential learning and advocacy—to ensure graduates’ skills are effectively applied and aligned with Nepal's evolving health priorities
		“The curriculum should be regularly updated to reflect current government systems, technologies, and national health priorities in Nepal. Students need to learn the practices actually implemented in the field to reduce the gap between theoretical knowledge and practical application.”—P5	
**6. Hope and future vision**	Educational reforms, expanded local roles, proactive professional initiatives, resilience, and engagement	“Even if the system is weak, professionals who are proactive, creative, and willing to learn independently will find opportunities.”—P8	Professionals emphasize that proactive, adaptable graduates can navigate systemic weaknesses, whereas local government recognition creates meaningful roles; despite challenges, they demonstrate agency, seeing opportunities to innovate, specialize, and shape Nepal's public health sector
		“I believe that the future of public health in Nepal depends on local governments realizing the importance of trained professionals. If public health officers are recruited at the ward and municipal level, graduates will have meaningful roles, and the field will grow.”—P10	

### 3.1 Navigating a Narrow Job Market

The participants described a public health job market characterized by scarcity and uncertainty, requiring persistence and strategic skill development. For example, P1 highlighted both the existence of opportunities and the difficulty of securing them:

There are both positives and negatives. Opportunities exist in NGOs, government vacancies, and academia, such as teaching, research, and community projects, but not all graduates secure employment … if 100 students graduate, many may remain without a job. (P1)

This reflects a broader sense of uncertainty, where students require proactive effort beyond academic achievement to secure employment. P3 emphasized the importance of aligning skills with market demands:

Finding a job is challenging. It took me a year to secure employment after graduation, but I kept applying. The beginning is difficult, yet persistence pays off. Development of skills aligned with job requirements is essential for success. (P3)

Some participants reported that competition with more qualified peers shaped career decisions. P7 stressed that when positions intended for BPH graduates are open, they often see competition from MPH students or more highly qualified candidates willing to work for less:

Even when vacancies are open for BPH graduates, MPH graduates often apply, and we miss out because those with higher qualifications and skills are willing to take the job at a lower cost than their credentials suggest. (P7)

Collectively, these perspectives indicate that PHPs navigate a labor market that is both competitive and unpredictable, prompting some graduates to pursue further education as a strategy to improve employability. For instance, P6 shared:

Owing to difficulty in job, people prefer joining masters …. The reason behind joining masters is to get a job easily. (P6)

These insights suggest that career navigation is not only about academic preparation but also about strategic persistence, skill development, and leveraging networks.

### 3.2 Struggling With Practical Gaps

Graduates reflected on a disconnect between theoretical knowledge and practical competence in public health. For instance, P9 noted that despite studying project management in theory, applying these skills in real‐world settings was challenging:

During our study, we focused mostly on books and assignments … when I joined a local NGO, I realized that I did not know how to manage a public health project, even though we studied it in theory. Universities say we are ready, but reality is different. (P9)

The absence of mentorship contributed to feelings of unpreparedness and uncertainty. P18 explained:

After graduation, I didn't know where to begin. With mentorship from someone to guide me … on skill development and job search, I would have felt more confident. Instead, I felt lost and unprepared to face the professional world. (P18)

Social capital and personal connections can influence job prospects, leaving those without networks frustrated and sometimes redirecting their careers. P10 observed:

NGOs and INGOs do have vacancies, but in our context, preference is often given to those with connections … NEPAL MA RAMRO LAI BHANDA PANI HAMRO LAI PRIORITY DIYINCHA …. Many public health graduates feel frustrated. Several of my friends switched fields, saying that without connections, studying public health feels pointless. (P10)

These narratives suggest that practical skill development is inconsistently integrated into formal education, and graduates often rely on personal initiative, networks, and mentorship to bridge the gap.

### 3.3 Barriers to Job Placement

The participants highlighted structural and systemic barriers to employment. P11 and P16 describe limited vacancies, preferential recruitment, and a lack of accountability in universities and employers, which collectively disadvantage even competent graduates.

P11 highlighted the disconnect between academic preparation and workforce expectations:

The major problem is that universities focus only on teaching, not on practical skills or accountability, resulting in graduates who are poorly prepared for the workforce. (P11)

Similarly, P16 described how personal connections influenced hiring decisions:

Competent candidates miss out on job opportunities owing to limited vacancies and unfair recruiting, as employers often prioritize their acquaintances. (P16)

These challenges contributed to feelings of demoralization among graduates. P15 explained:

Even after graduation, you constantly feel stressed… I was lucky to find a job when some of my friends were even thinking about leaving public health entirely… waiting for job is so demoralizing. (P15)

Disparities in practical training, including internship availability, further reinforced inequities. P9 used a metaphor to convey the abrupt transition from education to professional life without adequate preparation:

At my university, there was a provision for internships, and some of my friends secured jobs in the same institutions where they interned. Friends from other universities, however, only graduated with certificates‐ they had no internships, no networking, and limited skill development. It felt like “throwing someone who can't swim into the sea.” (P9)

Collectively, these narratives highlight that structural gaps, unequal access to practical experiences, and preferential hiring practices significantly shape graduates’ career trajectories, creating challenges that extend beyond individual effort or merit.

### 3.4 Foundations Misaligned With Practice

Participants reflected on the disconnect between academic curricula and professional practice, highlighting outdated syllabi and limited practical training. P15 described the difficulty of accessing practical learning opportunities:

We had to fight with the department just to get a training program on data analysis. Without such practical training, it's hard to keep up with global public health standards. (P15)

P13 reflected on the lag between course content and real‐world public health systems:

The syllabus has not changed for decades. Even when government systems updated their processes, our courses were the same as before. We were not prepared for current field realities because the curriculum lagged behind actual public health practice. (P13)

P11 illustrated how theoretical knowledge often fails to align with workplace requirements:

In our course, we studied biostatistics and planning processes, but in my job, I never use them. The government system uses tools such as DHIS, which are not part of our curriculum. What we study and what is required in practice don't match, and adapting to the real system depends entirely on oneself. (P11)

Concerns were also raised about institutional oversight and competency assurance. P1 questioned whether universities and regulatory bodies adequately equip graduates for professional demands:

We have NHPC for quality control. They conduct licensing exams, where only around half of the students pass … the question arises about students’ competencies … competencies come from universities, and finally, the question arises about universities themselves‐ whether a university is providing the right education and training. (P1)

These narratives collectively indicate a systemic misalignment between foundational education and field realities, requiring both curricular updates and stronger institutional accountability to ensure graduates are prepared for contemporary public health practice.

### 3.5 Overcoming the Challenges

Despite structural and educational limitations, participants identified pathways to enhance professional readiness and career progression. P14 emphasized the potential of creating dedicated public health positions at the local level, which could optimize graduates’ skills and reduce dependence on other health cadres.

The government should allocate specific posts for public health graduates at the Palika level. This will reduce overreliance on other health cadres and ensure that graduates’ skills are fully utilized in community health management. (P14)

Several participants emphasized the importance of updating curricula and providing mentorship to strengthen workforce preparedness. P5 underscored the need for alignment between education and current government systems:

The curriculum should be regularly updated to reflect current government systems, technologies, and national health priorities in Nepal. Students need to learn the practices actually implemented in the field to reduce the gap between theoretical knowledge and practical application. (P5)

P11 described how recognition, structured mentorship, and opportunities for professional development could motivate early‐career PHPs to remain engaged in the sector:

I sometimes feel that the work we do is undervalued, especially as early‐career professionals. Recognition, mentorship, and structured opportunities for improvement could motivate us to stay and perform better in the public health sector. (P11)

Finally, P17 highlighted the role of regulatory bodies, such as the NHPC and MEC, to establish competency‐based standards and clear career pathways, suggesting that institutional support is essential to professional growth.

I think the NHPC and MEC should step in to create policies that actually recognize and support public health graduates. They should make career pathways clear, set competency‐based standards, and ensure that our education and skills are valued in the system, not just for doctors and nurses but for all public health professionals. (P17)

Collectively, these perspectives indicate that meaningful reform requires both systemic changes—such as curriculum updates, structured career pathways, and institutional support—and individual advocacy to navigate the evolving public health landscape.

### 3.6 Hope and Future Vision

Despite the challenges described earlier, participants expressed cautious optimism about the future of public health in Nepal. Several pointed to emerging institutional reforms and evolving professional roles that could strengthen the field. For example, P9 noted that MEC‐led reforms have opened new educational opportunities:

Over the past few years, MEC has conducted entrance exams for postgraduate studies, requiring students to pass before studying in Nepal or abroad. While this may delay those pursuing studies abroad and incur additional costs, the MEC system has created new opportunities, including integrated scholarships across universities. It has also facilitated the bonding of graduates with the Nepali health system, supporting workforce retention and providing exposure to government operations. (P9)

Participants also emphasized the potential role of local governments in expanding employment opportunities for trained PHPs. P10 highlighted the potential of local government recruitment to expand professional roles:

I believe that the future of public health in Nepal depends on local governments realizing the importance of trained professionals. If public health officers are recruited at the ward and municipal level, graduates will have meaningful roles, and the field will grow. (P10)

At the same time, some participants suggested that individual initiative and adaptability are important for navigating a system that still faces structural limitations. P8 emphasized describing how proactive, innovative professionals can create opportunities despite systemic weaknesses:

Even if the system is weak, professionals who are proactive, creative, and willing to learn independently will find opportunities. (P8)

Similarly, P16 described how entrepreneurial approaches, such as establishing health consultancies or community health initiatives, could enable professionals to create opportunities rather than relying solely on formal employment structures:

Public health professionals can start their own initiatives‐ health consultancy, wellness centers, or community health programs. Those who are proactive can create jobs instead of waiting for positions in government or private sectors. (P16)

These reflections suggest that resilience, creativity, and engagement with institutional reforms are central to shaping a sustainable and meaningful public health workforce.

## 4 Discussion

This study explored the experiences of early‐career PHPs in Nepal and their perceptions of employment opportunities, professional preparation, and workforce challenges. The findings indicate a clear misalignment between academic preparation and professional realities. Graduates described a constrained job market, gaps in practical skills, and structural barriers to employment, including limited opportunities and perceived non‐merit‐based recruitment practices. These challenges often reduced motivation to remain in the public health sector and encouraged some graduates to pursue further education or shift career paths. However, participants also demonstrated resilience by proposing strategies such as networking, skill development, and entrepreneurial initiatives to navigate these constraints.

These findings are consistent with broader global discussions about the evolving role and expectations of PHPs. Public health systems increasingly require professionals who can operate across complex and interdisciplinary environments, addressing emerging challenges such as health inequities, environmental change, and global health threats [1, 7]. However, studies from several countries have identified persistent gaps between public health education and workforce demands, particularly in relation to applied competencies and practical experience [9, 10, 16]. For example, analyses of public health training programs have highlighted the need to better align curricula with employer expectations and real‐world practice environments [14, 21]. In Nepal specifically, earlier assessments of graduate public health education similarly identified concerns regarding the balance between theoretical instruction and practical skill development [24]. The experiences described by participants in this study therefore reinforce existing evidence suggesting that educational reforms may be necessary to ensure that graduates are adequately prepared for professional roles within complex health systems.

An important finding emerging from this study is the emphasis participants placed on structural barriers to employment, including limited career pathways and concerns regarding transparency in recruitment processes. Although workforce challenges are not unique to Nepal, they may be particularly pronounced in resource‐constrained health systems where public health positions are limited and institutional frameworks for workforce planning are still evolving [35]. At the same time, the participants’ accounts of self‐directed initiatives and entrepreneurial approaches highlight an important dimension of agency within the public health workforce. Such strategies align with broader calls for PHPs to act as agents of social change and innovation within their communities [19]. Encouraging these forms of professional initiative may help expand the role of PHPs beyond traditional government positions, particularly in contexts where formal employment opportunities are limited.

Alongside the challenges identified, the literature points to actionable opportunities to strengthen the public health workforce through more effective structuring of entry and progression pathways. Evidence indicates that clearer and more accessible recruitment mechanisms, greater transparency in roles and career trajectories, and stronger institutional linkages between training programs and employers can improve the transition from education to employment and better align workforce supply with system needs [31]. In addition, expanding and formalizing pathways such as internships, fellowships, and cross‐sector entry routes is particularly important, given that many early‐career professionals enter public health from non‐traditional backgrounds [32]. When supported by structured mentorship and leadership development, these approaches can contribute to improved retention and workforce sustainability. In the context of Nepal, such strategies offer practical avenues to address the structural constraints highlighted in this study while strengthening workforce adaptability and long‐term capacity.

The findings also have implications for strengthening public health education and workforce development. Educational institutions could consider incorporating more applied and competency‐based training approaches, including field‐based learning, multidisciplinary collaboration, and structured internships. Similar reforms have been recommended internationally to ensure that public health graduates possess the skills necessary for policy engagement, program implementation, and community‐based interventions [18, 23]. In addition, supporting early‐career PHPs through mentorship, continuing professional development, and clear career pathways may help improve workforce retention and job satisfaction [27]. Strengthening accreditation and quality assurance mechanisms for public health programs could further contribute to ensuring consistent training standards and alignment with workforce needs [59].

Reflexive methods were central to this analysis. Ongoing journaling and discussions with colleagues helped challenge assumptions and maintain a focus on participants’ experiences [51]. Semi‐structured interviews provided in‐depth insights, although the researcher's dual role as both insider and outsider may have affected what participants shared. Reflexive practices helped ensure that the analysis highlighted participants’ actions and choices rather than presenting systemic issues as fixed or inevitable.

Several limitations should be considered when interpreting the findings of this study. First, the sample consisted of public health graduates with up to 5 years’ professional experience since their most recent degree, which means the findings primarily reflect early‐career perspectives and may not capture the experiences of more senior professionals. However, qualitative research prioritizes depth of understanding rather than statistical representation, and the sample size was considered sufficient based on the concept of information power in qualitative studies [54]. Second, participants were recruited purposively, and those with strong views about workforce challenges may have been more likely to participate, potentially introducing response bias. Third, the reliance on self‐reported experiences implies that some perspectives reflect subjective perceptions and may not fully reflect institutional or policy realities. Finally, the absence of perspectives from other stakeholders, including educators, policymakers, and senior professionals, constrains the comprehensiveness of systemic critique.

Future research could build on these findings by examining workforce challenges from multiple stakeholder perspectives, including policymakers, academic institutions, and employers within the health system. Comparative studies across provinces or between countries with similar health system structures may also provide further insights into effective strategies for strengthening the public health workforce. In addition, quantitative labor market analyses could help clarify the availability of public health positions and identify structural factors influencing employment opportunities.

This study highlights the need for stronger alignment between public health education, workforce governance, and labor market realities in Nepal. Addressing gaps between academic preparation and professional practice, improving transparency in recruitment processes, and strengthening institutional support for early‐career professionals may be critical for developing a resilient public health workforce capable of responding to evolving health challenges.

## 5 Conclusion

This study underscores the urgent need to reimagine Nepal's public health ecosystem by aligning academic preparation with professional realities. Strengthening curricula with competency‐based and practical training, integrating modern teaching technologies, providing mentorship and field exposure, ensuring transparent and adequate employment opportunities, and implementing supportive policies can empower public health students, graduates, and professionals to thrive throughout their career trajectory. Such reforms not only enhance workforce resilience and career satisfaction but also strengthen the capacity to improve population health outcomes in resource‐constrained contexts.

## Author Contributions

Ankit Acharya conceptualized the study, designed the methodology, conducted and supervised the data collection and analysis, drafted the manuscript, approved the final version, and was accountable for the contributions made. Romika Neupane, Bibekti Nepal, Liberty Shrestha, and Princy Bhatta contributed to the data collection, analysis, and interpretation; contributed to manuscript revision; approved the final version; and were accountable for their contributions.

## Funding

The authors have nothing to report.

## Ethics Statement

Ethical approval was obtained from the Institutional Review Committee at Nobel College, Pokhara University (Ref no.: EPHIRC036/2022).

## Conflicts of Interest

The authors declare no conflicts of interest.

## Supporting information




**Supporting File:** puh270327‐sup‐0001‐Appendix.docx.

## Data Availability

The datasets used and/or analyzed during the current study are available from the corresponding author upon reasonable request.
