# Supplementary material for: Challenges and Opportunities of Public Health Professionals in Nepal: A Reflexive Thematic Analysis
Source: Public Health Chall. 2026 Aug 26;5(3):e70327. doi: 10.1002/puh2.70327 (PMC13507930; doi:10.1002/puh2.70327)
Supplement: Supplementary file 1 [file PUH2-5-e70327-s001.docx]

# S1 Appendix. In-Depth Interview Guideline

**Demographics:**

Name:

Age:

Gender:

Job status with year of experience:

Permanent and temporary address:

1. How do you see the availability of job opportunities (as well as further educational opportunities) after graduation in Public Health (PH) in Nepal? *(Probe: individual experience/ general perception; how do you see the recent preference of fresh graduates to obtain postgraduate degrees rather than obtaining field experiences for few years)*
2. In your opinion, what are the common challenges faced by PH graduates in Nepal?
3. Why do you think PH graduates are having difficulties in job placement after graduation? *(Probe: Inadequate knowledge and skills, no prior experiences, nepotism and less governmental vacancies)*

*4.*What is the reason behind these difficulties? *(Probe: theory-based teaching learning, inadequately trained faculties, non-availability of learning resources, poor use of technology, inadequate skill-oriented trainings, inadequate skills and competencies of students)*

5. What can be done to overcome these challenges and what are your recommendations to concerned stakeholders?
6. Is there anything you would like to add? *(Probe: How hopeful are you regarding the future of public health and public health graduates in Nepal?)*
